# Supplementary material for: A short intrinsically disordered domain of MCPyV ALTO regulates TBK1 signaling during MCPyV infection
Source: J Virol. 2025 Nov 24;99(12):e01341-25. doi: 10.1128/jvi.01341-25 (PMC12724334; doi:10.1128/jvi.01341-25)
Supplement: Supplemental material — Tables S1 and S2; Fig. S1 to S9. [file jvi.01341-25-s0001.pdf]

Supplemental Table 1: Cloning Primer Sequences

Primer	Sequence
Src NheI F	5'- GATCGCTAGC ATG GGTAGCAACAAGAGCAAG-3'
Src XhoI R	5'-GATCCTCGAGCC GAGGTTCTCCCCGGGC-3'
ALTO NheI R	5'-CAGTGCTAGCTCAGATCTGTTGCTTGATACGATG-3'
Ψ XhoI F	5'-CAGTCTCGAGCGGTCAGGACCCCGAGTCATTG-3'
χ XhoI F	5'-CAGTCTCGAGCGGTATGGGCCCAAGC-3'
Φ XhoI F	5'-CAGTCTCGAGCGGTCTCGACCCAGTGGCC-3'
υ XhoI F	5'-CAGTCTCGAGCGGTAGAATGGCACCAGGACG-3'
τ XhoI F	5'-CAGTCTCGAGCGGTCCACAAGAGGCCAGGC-3'
σ XhoI F	5'-CAGTCTCGAGCGGTCCAAGCCCACCAAGGC-3'
ρ XhoI F	5'-CAGTCTCGAGCGGTTTGCTGTTTCTGCTCATCTTC-3'
ALTO XhoI F	5'-CAGTCTCGAGCGGTGGCCCCTGAACAGCAAG-3'
ALTOΔTM NheI R	5'-CAGTGCTAGCTCACTTTTCGGTGCGCTTCTG-3'
LIT XhoI F	5'-CAGTCTCGAGCGGTAGAAGGCACCAGGAC-3'
LIT NheI R	5'-CAGTGCTAGCTCACGGTGGGCTGCG-3'
delLIT Bpu10I F	5'-CAGTCCTGAGCCACAAGAGGCCAGG-3'
delLIT Bpu10I R	5'-CAGTGCTCAGGCAAGATTCCAGATAGAC-3'
ALTO BamHI F	5'-CAGGGATCCGGCCCACTGAACAGCAAG-3'
ALTO150/LIT XhoI R	5'-CAGTCTCGAGTCACGGTGGGCTGCG-3'
ALTOdelLIT AgeI F	5'-ACTGACCGGTACCATGGGCCCACTGAACAGC-3'
ALTOdelLIT MluI R	5'-TGTCACGCGTTCATTGCTTGATAACGATGTAGAGC-3'
LgBiT-LIT BamHI F	5'-CAGTGGATCCATGGTCTTCACACTCGAAG-3'
LgBiT-LIT EcoRI R	5'-CAGTGAATTCTCACGGTGGGCTGC-3'

Supplemental Table 2: qPCR Primer Sequences

Primer	Sequence
MCPyV NCRR F	5’-TAGGCAGCCAAGTTGTGGTTA-3’
MCPyV NCRR R	5’-CGTCTCCCTCCCAAACAGAAA-3’
Genomic GAPDH F	5’-GGCCCTGACAACTCTTTTCATCTT-3’
Genomic GAPDH R	5’-CAACTGTGAGGAGGGGAGATTC-3’

Figure S1

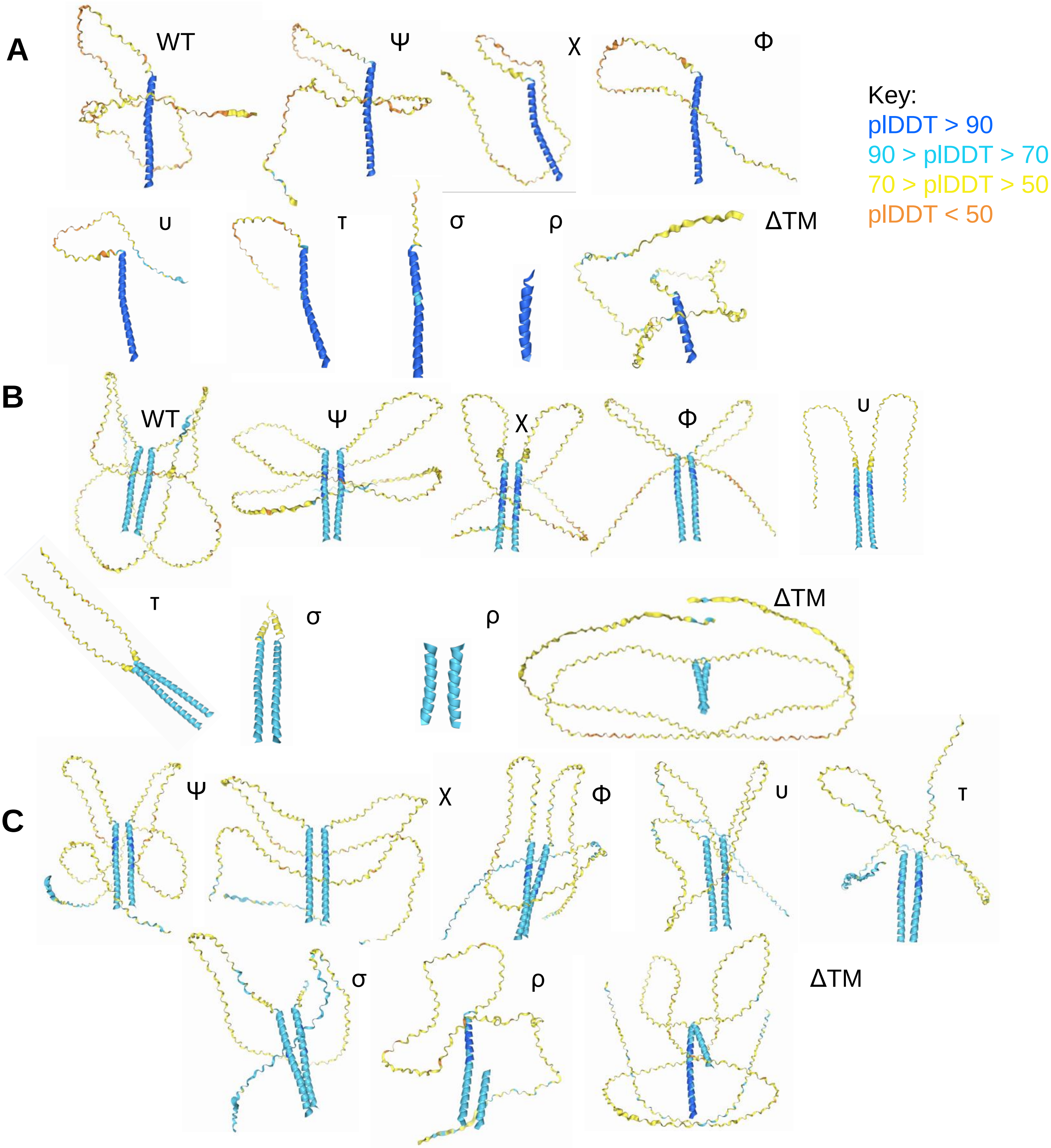

A
WT
Ψ
χ
Φ
υ
τ
σ
ρ
ΔTM
Key:
plDDT > 90
90 > plDDT > 70
70 > plDDT > 50
plDDT < 50
B
WT
Ψ
χ
Φ
υ
τ
σ
ρ
ΔTM
C
Ψ
χ
Φ
υ
τ
σ
ρ
ΔTM

Figure S2

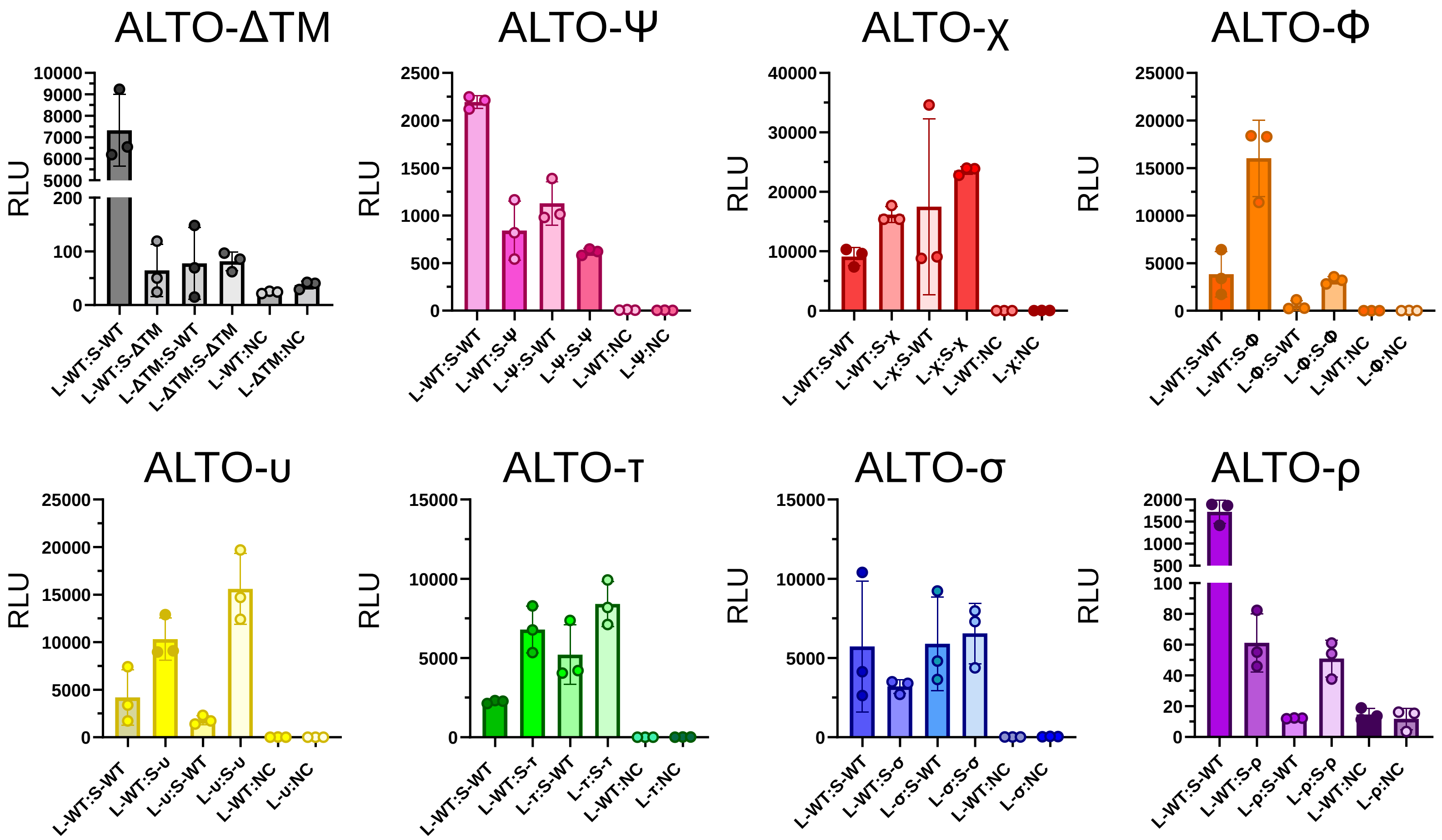

ALTO-ΔTM
ALTO-Ψ
ALTO-χ
ALTO-Φ
ALTO-υ
ALTO-τ
ALTO-σ
ALTO-ρ
RLU
L-WT:S-WT
L-WT:S-ΔTM
L-ΔTM:S-WT
L-ΔTM:S-ΔTM
L-WT:NC
L-ΔTM:NC
L-WT:S-Ψ
L-Ψ:S-WT
L-Ψ:S-Ψ
L-Ψ:NC
L-WT:S-χ
L-χ:S-WT
L-χ:S-χ
L-χ:NC
L-WT:S-Φ
L-Φ:S-WT
L-Φ:S-Φ
L-Φ:NC
L-WT:S-υ
L-υ:S-WT
L-υ:S-υ
L-υ:NC
L-WT:S-τ
L-τ:S-WT
L-τ:S-τ
L-τ:NC
L-WT:S-σ
L-σ:S-WT
L-σ:S-σ
L-σ:NC
L-WT:S-ρ
L-ρ:S-WT
L-ρ:S-ρ
L-ρ:NC

Figure S3

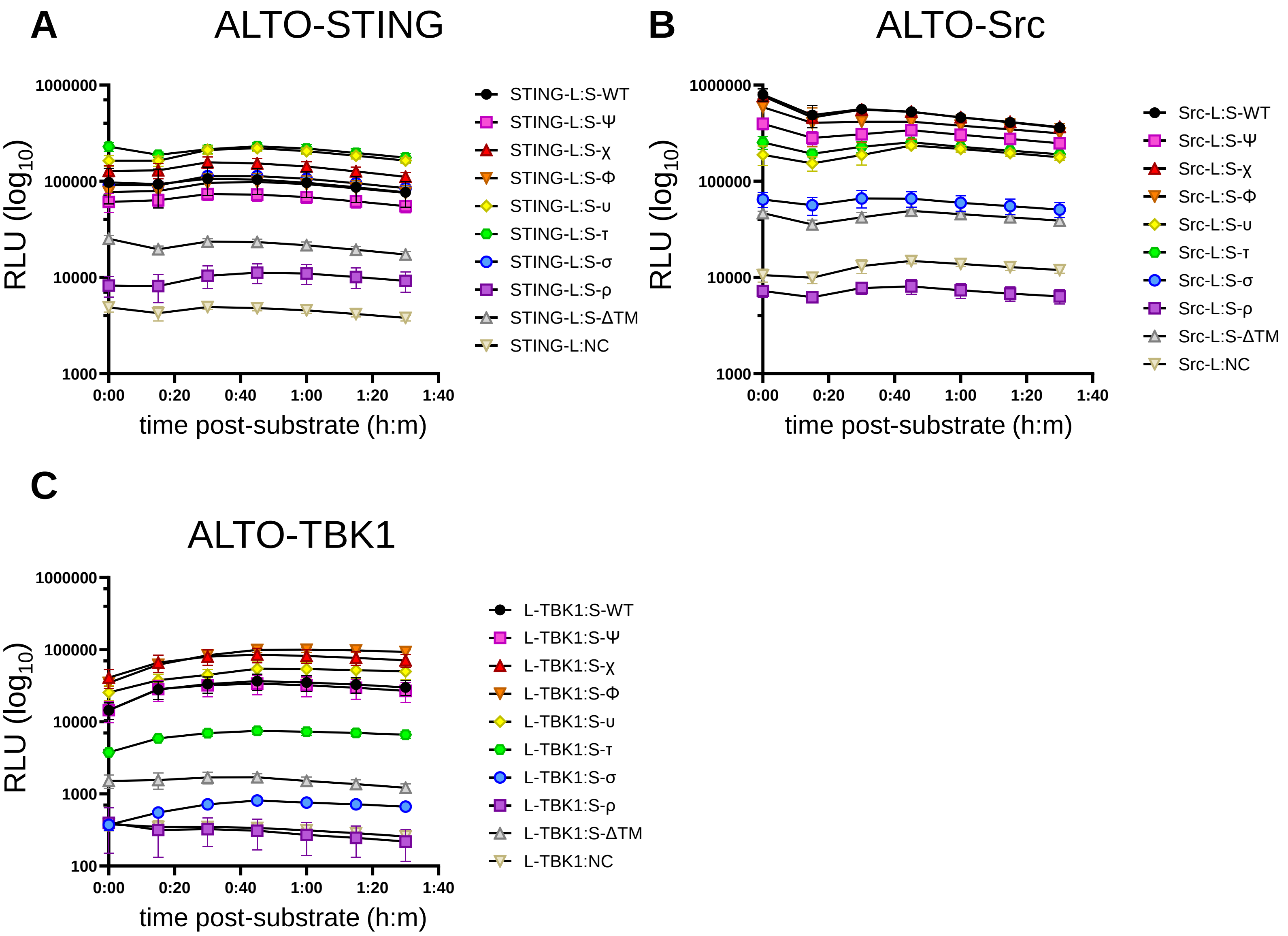
A
ALTO-STING
RLU (log10)
1000000
100000
10000
1000
0:00
0:20
0:40
1:00
1:20
1:40
time post-substrate (h:m)
STING-L:S-WT
STING-L:S-Ψ
STING-L:S-χ
STING-L:S-Φ
STING-L:S-υ
STING-L:S-τ
STING-L:S-σ
STING-L:S-ρ
STING-L:S-ΔTM
STING-L:NC
B
ALTO-Src
RLU (log10)
1000000
100000
10000
1000
0:00
0:20
0:40
1:00
1:20
1:40
time post-substrate (h:m)
Src-L:S-WT
Src-L:S-Ψ
Src-L:S-χ
Src-L:S-Φ
Src-L:S-υ
Src-L:S-τ
Src-L:S-σ
Src-L:S-ρ
Src-L:S-ΔTM
Src-L:NC
C
ALTO-TBK1
RLU (log10)
1000000
100000
10000
1000
100
0:00
0:20
0:40
1:00
1:20
1:40
time post-substrate (h:m)
L-TBK1:S-WT
L-TBK1:S-Ψ
L-TBK1:S-χ
L-TBK1:S-Φ
L-TBK1:S-υ
L-TBK1:S-τ
L-TBK1:S-σ
L-TBK1:S-ρ
L-TBK1:S-ΔTM
L-TBK1:NC

Figure S4

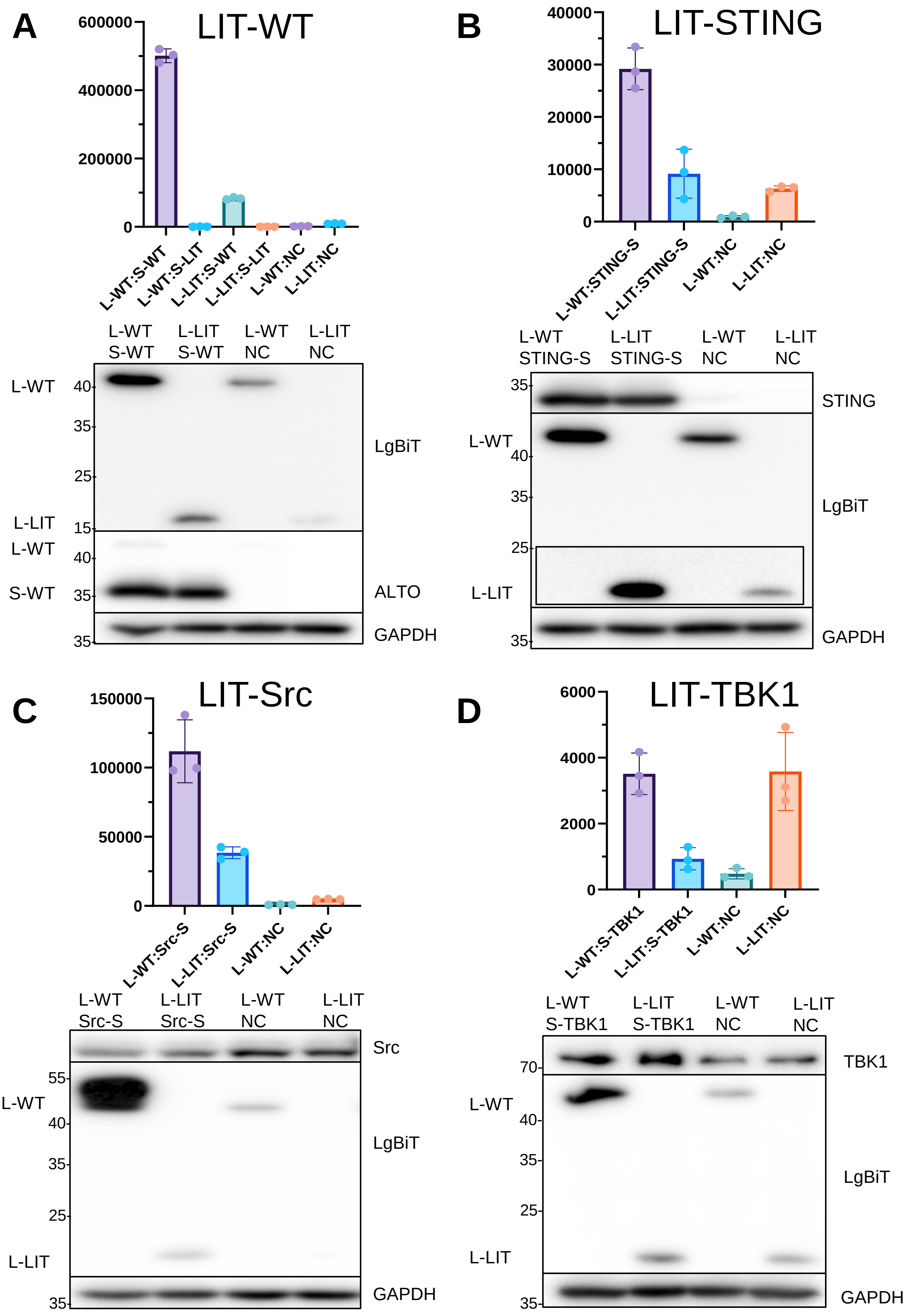

A
LIT-WT
600000
400000
200000
0
L-WT:S-WT
L-WT:S-LIT
L-LIT:S-WT
L-LIT:S-LIT
L-WT:NC
L-LIT:NC
L-WT S-WT
L-LIT S-WT
L-WT NC
L-LIT NC
L-WT
L-LIT
S-WT
40
35
25
15
LgBiT
ALTO
GAPDH
B
LIT-STING
40000
30000
20000
10000
0
L-WT:STING-S
L-LIT:STING-S
L-WT:NC
L-LIT:NC
L-WT STING-S
L-LIT STING-S
L-WT NC
L-LIT NC
STING
LgBiT
GAPDH
C
LIT-Src
150000
100000
50000
0
L-WT:Src-S
L-LIT:Src-S
L-WT:NC
L-LIT:NC
L-WT Src-S
L-LIT Src-S
L-WT NC
L-LIT NC
55
Src
LgBiT
GAPDH
D
LIT-TBK1
6000
4000
2000
0
L-WT:S-TBK1
L-LIT:S-TBK1
L-WT:NC
L-LIT:NC
L-WT S-TBK1
L-LIT S-TBK1
L-WT NC
L-LIT NC
70
TBK1
LgBiT
GAPDH

Figure S5

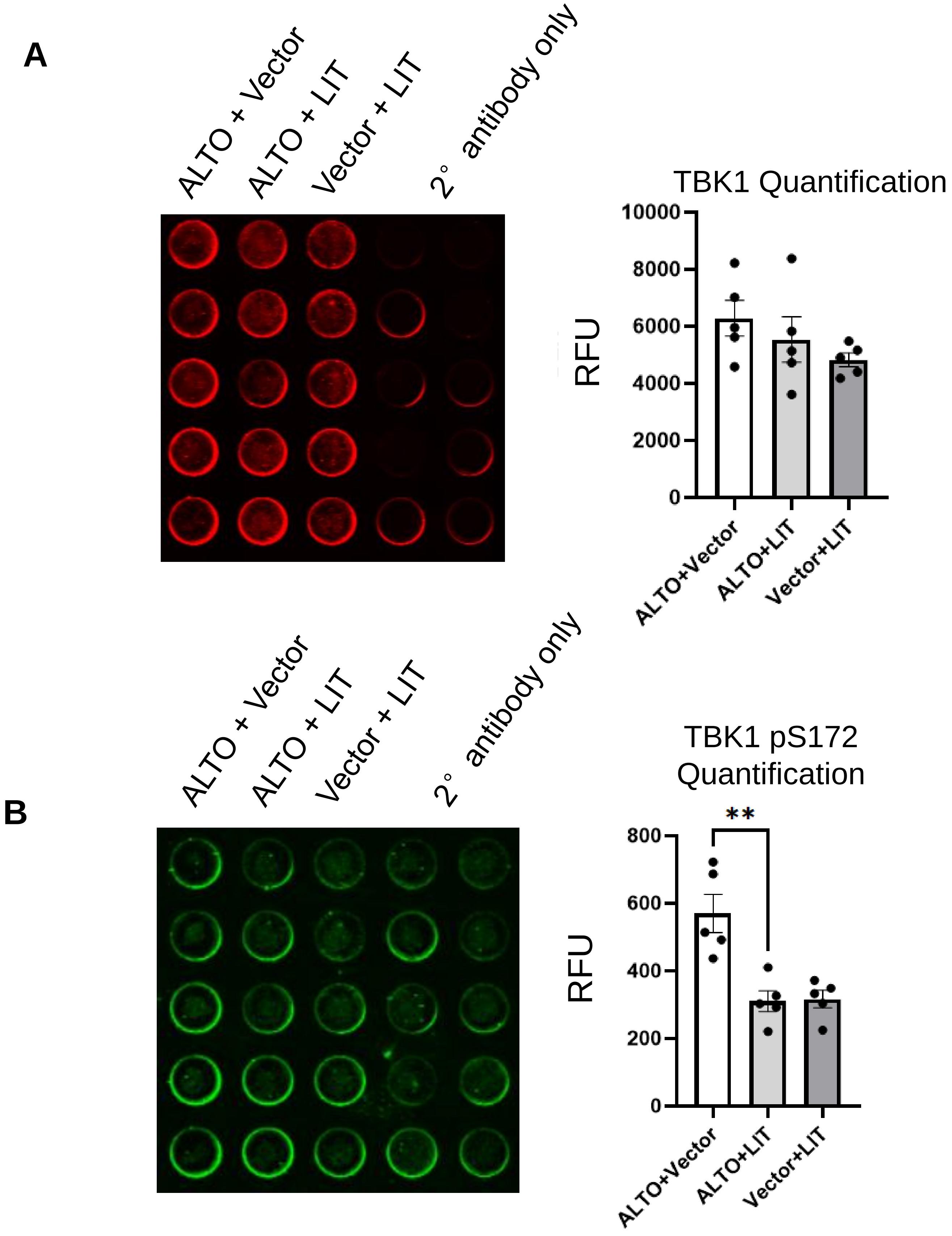

A
ALTO + Vector
ALTO + LIT
Vector + LIT
2° antibody only
TBK1 Quantification
RFU
10000
8000
6000
4000
2000
0
ALTO+Vector
ALTO+LIT
Vector+LIT
B
ALTO + Vector
ALTO + LIT
Vector + LIT
2° antibody only
TBK1 pS172 Quantification
**
RFU
800
600
400
200
0
ALTO+Vector
ALTO+LIT
Vector+LIT

Figure S6

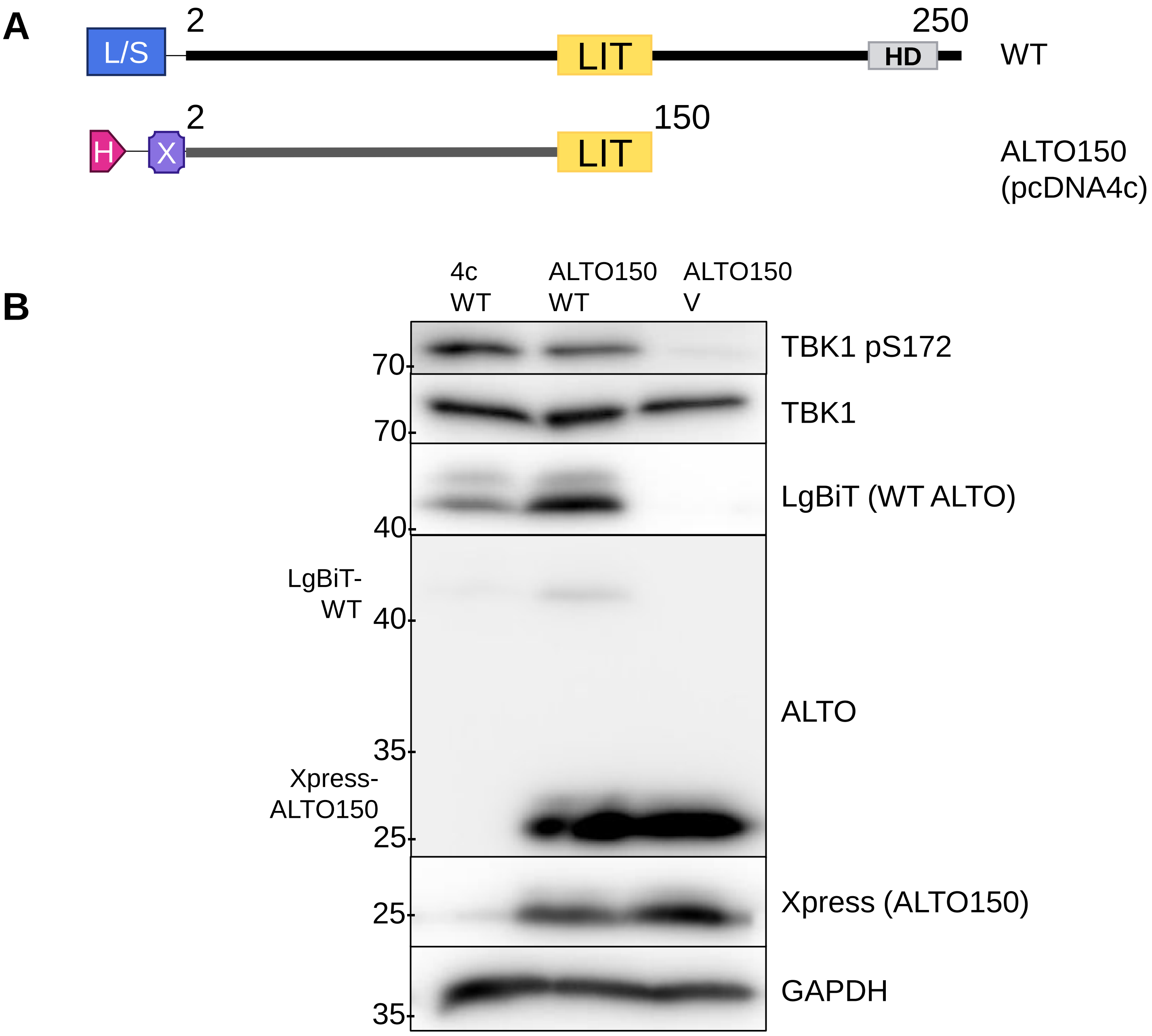
A
L/S
2
LIT
250
HD
WT
H
X
2
LIT
150
ALTO150
(pcDNA4c)
B
4c
WT
ALTO150
WT
ALTO150
V
70
TBK1 pS172
70
TBK1
40
LgBiT (WT ALTO)
LgBiT-
WT
40
ALTO
35
Xpress-
ALTO150
25
25
Xpress (ALTO150)
35
GAPDH

Figure S7

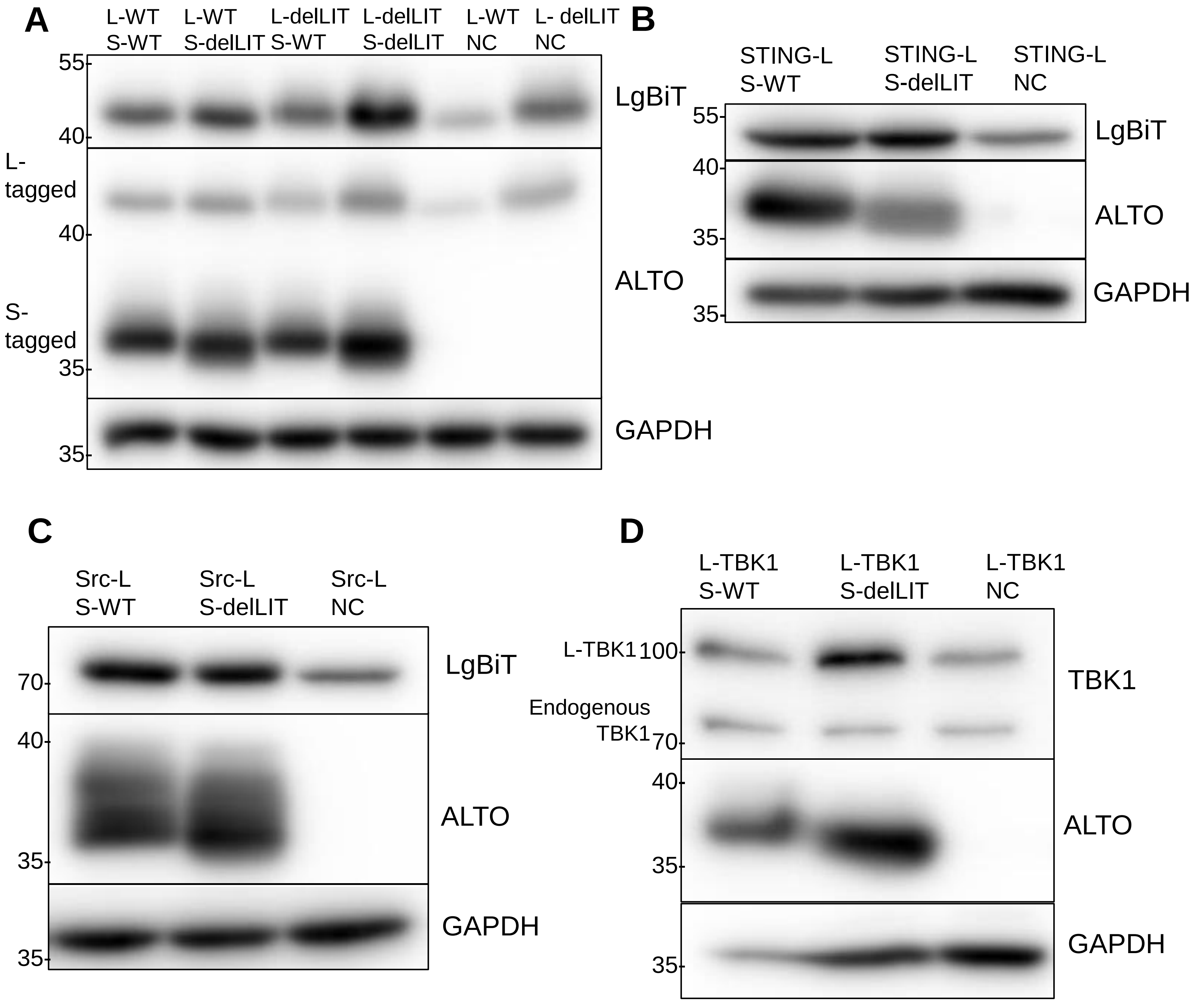

A
L-WT S-WT
L-WT S-delLIT
L-delLIT S-WT
L-delLIT S-delLIT
L-WT NC
L- delLIT NC
55
40
L-tagged
40
S-tagged
35
35
LgBiT
ALTO
GAPDH
B
STING-L S-WT
STING-L S-delLIT
STING-L NC
55
40
35
35
LgBiT
ALTO
GAPDH
C
Src-L S-WT
Src-L S-delLIT
Src-L NC
70
40
35
35
LgBiT
ALTO
GAPDH
D
L-TBK1 S-WT
L-TBK1 S-delLIT
L-TBK1 NC
L-TBK1
100
Endogenous TBK1
70
40
35
35
TBK1
ALTO
GAPDH

Figure S8

ELM Name & Description	Matched Sequence	Amino Acid Positions	Motif Type	Probability
**DOC_PP2A_B56_1**<br>Docking site for PP2A regulatory subunit B56	LEILPER	115-121	Docking	0.001458
**DOC_USP7_MATH_1**<br>USP7 MATH domain binding motif	PLRSL	142-146	Docking	0.01239
**LIG_14-3-3_CanoR_1**<br>Canonical Arg-containing phospho-motif mediating interaction with 14-3-3 proteins	RISSAMNHFP	126-135	Ligand binding	0.004477
	RPLRSLR	141-147	Ligand binding	
**LIG_LIR_Gen_1**<br>Canonical LIR motif binding to LC3 family proteins to mediate autophagy	EPVYLEILPERM	111-122	Ligand binding	0.003631
**MOD_GlcNHglycn**<br>Glycosaminoglycan attachment site	SSAM	128-131	Post-translational modification	0.01792
**MOD_PKA_2**<br>Secondary preference for PKA-type AGC kinase phosphorylation	GRISSAM	125-131	Post-translational modification	0.009458
**MOD_ProDKin_1**<br>Proline-Directed Kinase phosphorylation site	SLRSPPP	145-151	Post-translational modification	0.01543

Figure S9

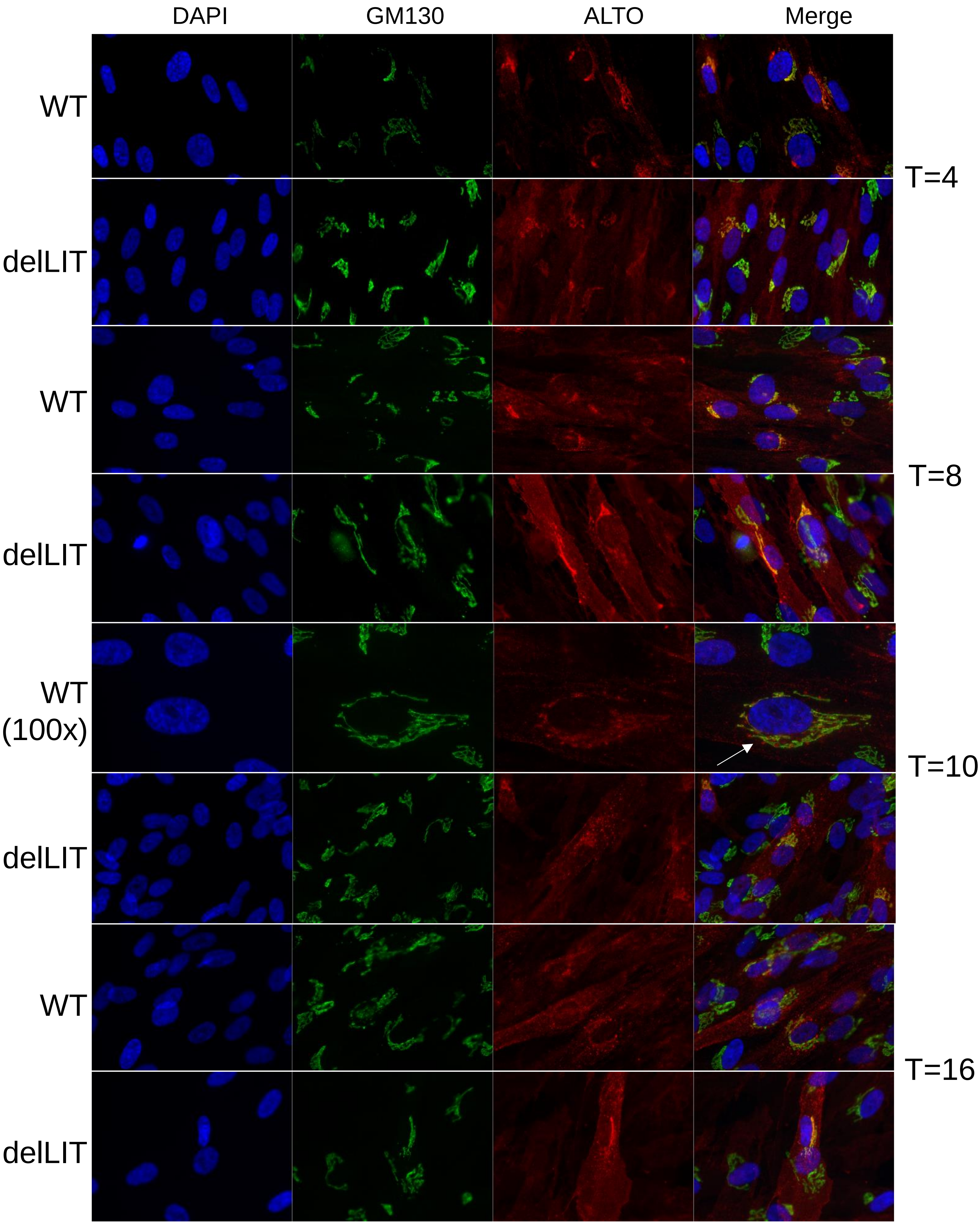

DAPI
GM130
ALTO
Merge
WT
delLIT
WT
delLIT
WT
(100x)
delLIT
WT
delLIT
T=4
T=8
T=10
T=16

Figure S1: Predictive tertiary structures of progressive ALTO truncation mutants. Amino acid sequences of progressive ALTO truncation mutants were used with the 2025.5.23 Update of the AlphaFold3 server platform (default settings, random seeding). All structures have a low-confidence intrinsically disordered N-terminal region, and a relatively higher-confidence C-terminal alpha helical structure, which is maintained through all truncations. All progressive ALTO truncations are shown in (A) monomeric, (B) homodimeric, and (C) WT ALTO-paired heterodimeric forms. Colors indicate AlphaFold calculated predicted local distance difference test (pIDDT) confidence scores according to the supplied key in (A).

Figure S2: Progressive ALTO truncation mutants retain homo-and hetero-oligomerization potential. Representative NanoBiT luminescence readings of HEK 293T cells transfected with pairs of plasmids carrying NanoBiT-tagged WT ALTO or a truncation mutant, or the Small BiT negative control construct. Luminescence was measured at 24hrs post-transfection and reported in Relative Luminescence Units (RLU). Points indicate biological replicate wells, bars indicate means, and error bars indicate standard deviations. L-protein and protein-S (and similar) indicate the named protein tagged with the LgBiT at its N-terminus or SmBiT at its C-terminus, respectively.

Figure S3: Kinetic curves of progressive ALTO truncation mutant interactions show relative interaction stability over time. Kinetic luminescence readings of HEK 293T cells 20hrs (ALTO-TBK1) or 24hrs (ALTO-STING and ALTO-Src) post-transfection with the indicated NanoBiT interaction pairs. Cells were monitored for 90 minutes after the addition of substrate, with readings recorded every 15min to determine the intensity and stability of protein-protein interactions. Individual points reflect average values of biological replicate wells; error bars indicate standard deviation across replicates at each time point; and trendlines indicate change between averages across timepoints. Single representative timepoints are highlighted in corresponding figures in Fig. 2.

Figure S4: Protein expression in LgBiT-LIT NanoBiT experiments is not sufficiently equal to WT to draw reliable interaction conclusions. Western blots and representative NanoBiT readings of HEK 293T cells co-transfected with tagged LIT and (A) WT ALTO, (B) STING, (C) Src, and (D) TBK1 at 20 hours (TBK1) or 24 hours (all others) post-transfection. Overexposed L-WT bands in (B) were covered to image the L-LIT bands (inset). Primary antibodies are indicated on the right; left-side labels differentiate bands visualized on the same blot.

Figure S5: Dual-channel in-cell western blot shows LIT inhibits ALTO-induced TBK1 autophosphorylation in HDFs. (A) Captured image (left) and relative fluorescence unit (RFU) quantification (right) of total TBK1 channel (700 nm) in HDFs transfected with WT ALTO and LIT or matching vector controls. (B) Captured image (left) and relative fluorescence unit (RFU) quantification (right) of TBK1 pS172 channel (800 nm). Total TBK1 was comparable across the three groups, whereas TBK1 pS172 signal was significantly lower in ALTO+LIT than in ALTO+Vector. Points reflect biological replicate wells, bars show mean ± SEM, n=5 wells/group; analysis shown is unpaired two-tailed t-test with Welch’s correction (ALTO+LIT vs ALTO+Vector), P = 0.0037 (significant).

Figure S6: ALTO150 is not sufficient to stimulate TBK1 autophosphorylation but is a partial dominant-negative. (A) Schematic diagram of LgBiT-tagged WT ALTO and 6* His-Xpress-tagged ALTO150 as expressed from the pcDNA4c vector (B) Western blots of lysates from HEK 293T cells 24 hours post-transfection with WT ALTO, ALTO150, or both. Right side labels indicate primary antibodies; left side labels distinguish multiple bands on the same blot.

Figure S7: Relatively equal protein expression across variants in ALTOdelLIT NanoBiT experiments supports functional conclusions. Western blots of lysates from HEK 293T cells transfected with indicated NanoBiT pairs, corresponding to Fig. 4. Lysates were harvested at 20hrs (ALTO-TBK1) or 24hrs (all others) post-transfection to assess relative protein expression

at the time of luminescence readings. Primary antibodies are listed on the right; labels on the left differentiate multiple bands on the same blot.

Figure S8: SLiMs do not explain the loss of function in ALTOdelLIT. Full-length amino acid sequence of ALTO was submitted to the ELM database (elm.eu.org) functional search prediction (cell compartment not specified, default parameters, Homo sapiens species context). Results have been filtered to display only motifs which are present within or immediately adjacent to the LIT domain. Probabilities provided by the ELM database reflect the likelihood of the motif to appear by random in any given protein sequence.

Figure S9: Longitudinal trafficking of WT ALTO and ALTOdelLIT. Representative immunofluorescence images of inALTO and inALTOdelLIT HDFs fixed at the indicated timepoints post-induction. All images taken with the 60x objective unless otherwise indicated. The WT cell shown at 100x at T=10 (white arrow) was particularly noteworthy for the apparent visualization of ALTO punctae leaving the Golgi.
